# Dietary Heme-Mediated PPARα Activation Does Not Affect the Heme-Induced Epithelial Hyperproliferation and Hyperplasia in Mouse Colon

**DOI:** 10.1371/journal.pone.0043260

**Published:** 2012-08-14

**Authors:** Noortje IJssennagger, Nicole de Wit, Michael Müller, Roelof van der Meer

**Affiliations:** 1 Top Institute Food and Nutrition, Wageningen, The Netherlands; 2 Nutrition, Metabolism and Genomics Group, Division of Human Nutrition, Wageningen University, Wageningen, The Netherlands; 3 Netherlands Nutrigenomics Centre, Wageningen, The Netherlands; Institut Jacques Monod, France

## Abstract

Red meat consumption is associated with an increased colon cancer risk. Heme, present in red meat, injures the colon surface epithelium by luminal cytotoxicity and reactive oxygen species. This surface injury is overcompensated by hyperproliferation and hyperplasia of crypt cells. Transcriptome analysis of mucosa of heme-fed mice showed, besides stress- and proliferation-related genes, many upregulated lipid metabolism-related PPARα target genes. The aim of this study was to investigate the role of PPARα in heme-induced hyperproliferation and hyperplasia. Male PPARα KO and WT mice received a purified diet with or without heme. As PPARα is proposed to protect against oxidative stress and lipid peroxidation, we hypothesized that the absence of PPARα leads to more surface injury and crypt hyperproliferation in the colon upon heme-feeding. Heme induced luminal cytotoxicity and lipid peroxidation and colonic hyperproliferation and hyperplasia to the same extent in WT and KO mice. Transcriptome analysis of colonic mucosa confirmed similar heme-induced hyperproliferation in WT and KO mice. Stainings for alkaline phosphatase activity and expression levels of Vanin-1 and Nrf2-targets indicated a compromised antioxidant defense in heme-fed KO mice. Our results suggest that the protective role of PPARα in antioxidant defense involves the Nrf2-inhibitor Fosl1, which is upregulated by heme in PPARα KO mice. We conclude that PPARα plays a protective role in colon against oxidative stress, but PPARα does not mediate heme-induced hyperproliferation. This implies that oxidative stress of surface cells is not the main determinant of heme-induced hyperproliferation and hyperplasia.

## Introduction

Colon cancer is a leading cause of cancer deaths in Western countries [1]. Epidemiological studies show that consumption of diets high in red- and processed meat is associated with the risk to develop colon cancer [2], [3]. Red meat is high in heme levels and it is shown that the addition of heme to diets of rats and mice induces hyperproliferation of colon epithelial cells [4], [5]. Hyperproliferation is a risk marker of colon cancer [6]. In contrast to the consumption of red meat, the consumption of white meat, which is low in heme, is not associated with an increased risk of colon cancer [7], [8]. In our recent studies we fed rodents a heme-rich diet or a control diet for 14 days [4], [5]. The heme diet increased the reactive oxygen species (ROS) levels, as well as cytotoxicity, of the colonic contents and induced damage to the surface epithelium. To compensate for the heme-induced damaged surface cells, hyperproliferation was initiated in the proliferative crypts and this eventually led to hyperplasia.

Microarray analysis of samples from whole colonic mucosa and from surface and crypt cells shows that heme regulates many stress and signaling-related genes in surface cells, and cell cycle genes specifically in crypt cells [5]. It is not known whether this heme-related surface to crypt signaling is caused by either oxidative stress or cytotoxic stress of surface cells. With regard to this, it may be of relevance that we found many PPARα target genes among the highest upregulated genes [5]. PPARα belongs to the superfamily of nuclear hormone receptors and known endogenous PPARα ligands are fatty acids and their derivatives such as oxidized fatty acids. Little is known, however, about the function of PPARα in colon. In the small intestine PPARα is mainly involved in lipid metabolism and absorption, but these processes are not likely to occur in the colon upon heme feeding. Based on literature, we hypothesize that PPARα is activated on a heme-rich diet to induce a protective mechanism against heme-induced oxidative stress and/or lipid peroxidation [9], facilitating the Nrf2-dependent antioxidant response. This potential PPARα-mediated protection against oxidative stress and/or lipid peroxidation could limit cell damage at the colonic surface epithelium and its compensatory hyperproliferation. This implies that knocking out PPARα would increase ROS-induced injury of surface cells and trigger the compensatory hyperproliferation of crypt cells.

The aim of this study was to investigate the role of PPARα in heme-induced hyperproliferation and hyperplasia in colon. Therefore, in our study wild-type (WT) mice were compared to PPARα knock-out (PPARα KO) mice on a control or heme-rich diet. Colonic cell damage and hyperproliferation were investigated and gene expression profiles were analyzed using microarrays.

## Materials and Methods

### Ethics Statement

The institutional and national guidelines for the care and use of animals were followed and the experiment was approved by the Local Committee for Care and Use of Laboratory Animals at Wageningen University.

### Animals and Diets

A breeding colony of pure-bred Sv129 PPARα knockout (KO) mice (129S4/SvJae) and corresponding wild-type (WT) mice (129S1/SvImJ) was purchased from Jackson Laboratory (Bar Harbor, ME) and bred at the animal facility of Wageningen University. Genotyping by performing quantitative PCR analysis for the ligand binding domain in exon 8 of the PPARα gene (primers: F: 5′-agaagttgcaggaggggatt-3′ and R: 5′-ttgaaggagctttgggaaga-3′), which was disrupted in the KO mice to disturb its function [10], verified that the mice were genuine PPARα KO mice. The WT and KO mice were housed individually in a room with controlled temperature (20–24°C), relative humidity (55%±15%) and a 12 h light dark cycle. To study whether PPARα plays a role in heme-induced hyperproliferation, 7–9 week old PPARα KO mice and wild-type mice received either a Westernized, purified, control diet (40 en% fat (mainly palm oil) low calcium (30 µmol/g)) or this diet supplemented with 0.5 µmol heme/g diet (Sigma-Aldrich Chemie, St. Louis) for 14 days (n = 6 per group, 4 groups) as previously described [11]. Body weight was recorded and feces were quantitatively collected during days 11–14, frozen at −20°C and subsequently freeze dried. After 14 days of intervention, the colon was excised, mesenteric fat was removed and the colon was opened longitudinally, washed in PBS, and cut into three parts. The middle 1.5 cm of the colon was formalin-fixed and paraffin embedded for histology. The remaining proximal and distal parts were scraped. Scrapings were pooled per mouse, snap-frozen in liquid nitrogen and stored at −80°C until further analysis.

### Fecal Analyses

Fecal water was prepared and cytotoxicity was measured for each mouse as previously described [5]. To determine lipid peroxidation products in the gut lumen Thiobarbituric Acid Reactive Substances (TBARS) in fecal water were quantified. The assay determines lipid peroxidation by quantifying the concentration of malondialdehyde (MDA) in fecal water [12]. Briefly, fecal water was diluted 4-fold with double-distilled water. To 100 µl of this dilution, 100 µl of 8.1% SDS and 1 ml of 0.11 mol/L 2,6-di-tert-butyl-p-cresol, 0.5% TBA in 10% acetic acid (pH 3.5) was added. To correct for background, TBA was omitted from the assay. TBARS were extracted, after heating for 75 minutes at 82°C, with 1.2 ml n-butanol. The absorbance of the extracts was measured at 540 nm. The amount of TBARS was calculated as MDA equivalents using 1,1,3,3,-tetramethoxypropane as standard.

### Immunohistochemistry

Hematoxylin and Eosin staining was performed to assess the morphology of the tissue. To stain proliferating cells, paraffin embedded colon sections of 5 µm were deparaffinized and stained with an anti-mouse Ki67 antibody as described previously [5]. Colonocytes from 15 well-oriented crypts (longitudinal section, displaying the total crypt) were counted per animal. These crypts were equally distributed over the middle 1.5 cm of the colon. A cell was scored Ki67 positive when the nucleus of the cell was distinctly brown. The number of Ki67 positive cells per crypt, the total number of cells per crypt and the labeling index (percentage of Ki67 positive cells per crypt) were determined. To determine alkaline phosphatase activity, colon tissue slides were deparaffinized and incubated with the alkaline-dye mixture (Alkaline phosphatase kit 85L2-1KT Sigma Aldrich) for 90 min at 37°C. Slides were rinsed with water and mounted.

### RNA Isolation

Total RNA was isolated by using TRIzol reagent (Invitrogen, Breda, The Netherlands) according to the manufacturer’s protocol. For microarray hybridization the isolated RNA was further column purified (SV total RNA isolation system Promega, Leiden, The Netherlands). RNA concentration was measured on a nanodrop ND-1000 UV-Vis spectrophotometer (Isogen, Maarssen, The Netherlands) and analyzed on an Agilent 2100 bioanalyzer (Agilent Technologies, Amsterdam, The Netherlands) with 6000 Nano Chips, according to the suppliers’ protocol. RNA was judged suitable for array hybridization only if samples exhibited intact bands corresponding to the 18S and 28S ribosomal RNA subunits, and displayed no chromosomal peaks or RNA degradation products (RNA Integrity Number >8.0).

### Array Hybridization and Microarray Data Analysis

One-hundred nanograms of RNA from each mouse (n = 6 per group) were used for whole-transcript cDNA synthesis with the Ambion WT expression kit (Applied Biosystems). Hybridization, washing and scanning of an Affymetrix GeneChip Mouse Gene 1.1 ST 24-array plate was carried out according to standard Affymetrix protocols on a GeneTitan instrument (Affymetrix). Quality control of the datasets was performed using Bioconductor packages [13] integrated in an on-line pipeline [14]. Due to insufficient quality, array results of 1 WT control mouse had to be excluded. Arrays were normalized using the Robust Multi-array Average method [15], [16]. Probe sets were defined according to Dai et al. [17]. Probe sets that satisfied the criterion of a False Discovery Rate (FDR) <1% (q-value <0.01) were considered to be significantly regulated. Genes with a signal intensity below 20 in both treatments were considered absent and excluded from further analyses. Array data were submitted to the Gene Expression Omnibus, accession number GSE37006.

Pathway analysis was performed using Ingenuity IPA Canonical Pathway Analysis (Ingenuity® Systems, May 2011, www.ingenuity.com). This analysis identifies the pathways from the Ingenuity Pathways Analysis library of canonical pathways that are most significant to a microarray data set. Fisher’s exact test was used to calculate a p-value determining the probability that the association between the genes in the dataset and the canonical pathway is explained by chance alone.

### Statistical Analysis

Data are presented as mean ± SEM. Differences between the mean values of the 4 groups were tested for main effects by a two-way ANOVA. A Bonferroni’s Multiple Comparison Test determined differences between groups. P-values <0.05 were considered significant.

## Results

### Heme Induced the Expression of PPARα Target Genes

**Figure 1:**
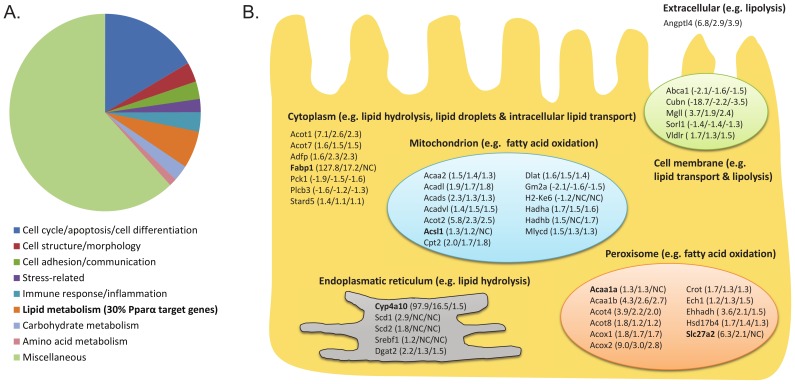
Effect of heme on PPARα target genes. **A.** Categorization of heme-induced differentially expressed genes (q<0.01 and signal intensity>20 in at least treatment) according to GO Biological Process annotation. Figure is based on results from IJssennagger et al. [5] showing that lipid metabolism-related gene expression is substantially influenced by heme. Thirty percent of these heme-induced lipid metabolism-related genes are PPARα target genes [18]. Miscellaneous contains processes with broad and thus unspecific biological process terms. **B.** Expression of PPARα target genes in enterocytes is mainly upregulated. Behind the gene the fold- changes are indicated from colonic scrapings from heme fed vs. control mice from resp. the previous experiment with C57Bl6J mice, current experiment WT SV129 mice and current experiment KO SV129 mice. In bold are PPARα targets of which no significant induction is seen in the KO mice.

Our recent studies show that the addition of heme to the diet of rats and C57Bl6J mice led to increased luminal reactive oxygen species (ROS) production and to increased cytotoxicity of the colonic luminal contents [4], [5]. This increase in heme-induced cytotoxicity damaged the surface cells in the colon and led to compensatory epithelial hyperproliferation [4], [5]. Microarray analysis of the colonic mucosa showed that most of the genes changed on the heme diet were involved in cell cycle/apoptosis/cell differentiation (Figure 1A, adapted from [5]). Furthermore, the study indicated that heme-induced hyperproliferation and hyperplasia was triggered by downregulating feedback inhibitors of proliferation, such as Wnt inhibitory factor 1 (Wif1), Interleukin-15 (IL-15), Indian Hedgehog (Ihh) and Bone morphogenetic protein 2 (Bmp2) in the surface epithelium [5].

Besides cell- and apoptosis-related genes, also lipid metabolism-related genes were highly regulated by the heme diet (Figure 1A). Of these lipid metabolism-related genes 30% were PPARα target genes as defined by Bunger et al. [18]. Amongst these PPARα targets Fabp1 and Cyp4a10 were the two highest upregulated genes with fold-changes of 128 and 98 respectively [5]. PPARα target genes and their change in expression in heme-fed C57Bl6J mice are summarized in Figure 1B (first fold-change listed between brackets). Next to gene expression of total colonic mucosa, gene expression levels of colon surface cells and colon crypt cells were separately determined by performing laser capture microdissection (LCM) [5]. The LCM study showed that most of the differentially expressed PPARα targets were present in the colonic surface cells (Gene Expression Omnibus, accession number GSE27849). No changes in PPARα targets were found in the lower crypt cells. This indicates that PPARα plays a role in the surface cells, where it possibly functions as a protective mechanism against e.g. oxidative stress and lipid peroxidation induced by the heme diet.

### Heme Induces Similar Lipid Peroxidation, Cytotoxicity, and Hyperproliferation in Colon of WT and KO Mice

As our previous studies show that heme injures the surface epithelium resulting in hyperproliferation, and we found that PPARα targets are highly induced in the surface epithelium, we now explored the potential role of PPARα in heme-induced hyperproliferation. We hypothesize that when there is no PPARα present in the colon, there is less protection against oxidative stress and/or lipid peroxidation. This attenuated protection would lead to an increase in heme-induced mucosal injury. As a damaged surface epithelium must trigger the compensatory hyperproliferation in the colonic crypt, the absence of PPARα would thus lead to an increased proliferation. To test this hypothesis, an experiment was performed in which PPARα knock-out (KO) mice and wild-type (WT) mice (both on a SV129 background) received either a control or a heme-rich diet for 2 weeks. After 2 weeks of diet-intervention, both the WT and the PPARα KO heme-fed mice had an increased cytotoxicity of the colon contents (Table 1). Luminal levels of lipid peroxidation products were determined by measuring TBARS in fecal water. TBARS were increased on the heme diet in both the WT and the PPARα KO compared to their control groups (Table 1). No significant differences between the heme-fed PPARα KO and the heme-fed WT mice were observed for bodyweight (23.0±0.8 and 21.8±1.0 g, respectively), cytotoxicity and TBARS. To measure colonocyte proliferation colon tissue was stained with an antibody against Ki67, a marker for proliferating cells. Cell counts revealed heme-induced increases in Ki67-positive cells per crypt as well as total number of cells per crypt in both the PPARα KO mice and WT mice, resulting in similar increases in the crypt labeling index (Table 1). Overall, there were no significant differences between the KO and the WT mice on the heme diet.

**Table 1:** Physiological changes induced by heme in colon of WT and PPARα KO mice.

	WT control	WT heme	KO control	KO heme
**Luminal contents**				
Cytotoxicity (% lysis)	0±0.8^a^	88.3±5.8^b^	1.8±2.7^a^	93.6±6.3^b^
TBARS (MDA equivalents, µmol/L)	28.3±6.8^a^	51.1±10.5^b^	28.3±6.4^a^	42.6±8.3^b^
**Mucosa**				
Total number of cells/crypt	41.1±2.0^a^	61.2±3.0^b^	45.4±2.2^a^	56.6±3.1^b^
Ki67 positive cells/crypt	15.8±1.2^a^	37.5±2.7^b^	17.7±1.9^a^	30.7±2.0^b^
Labeling index#	38.5±2.1^a^	60.9±2.6^b^	38.2±2.4^a^	54.1±1.6^b^

# Calculated as percentage Ki67 positive cells per crypt. Data are represented as mean ± SEM. Groups indicated with ‘a’ are significantly (*P*<0.05) different from ‘b’ by ANOVA with Bonferroni post-hoc testing. N = 6 per group, except for mucosa measurements where proliferation of one KO heme animal could not be determined due to poor tissue quality.

### Most PPARα Targets were also Induced in the KO Mice on the Heme Diet

**Figure 2:**
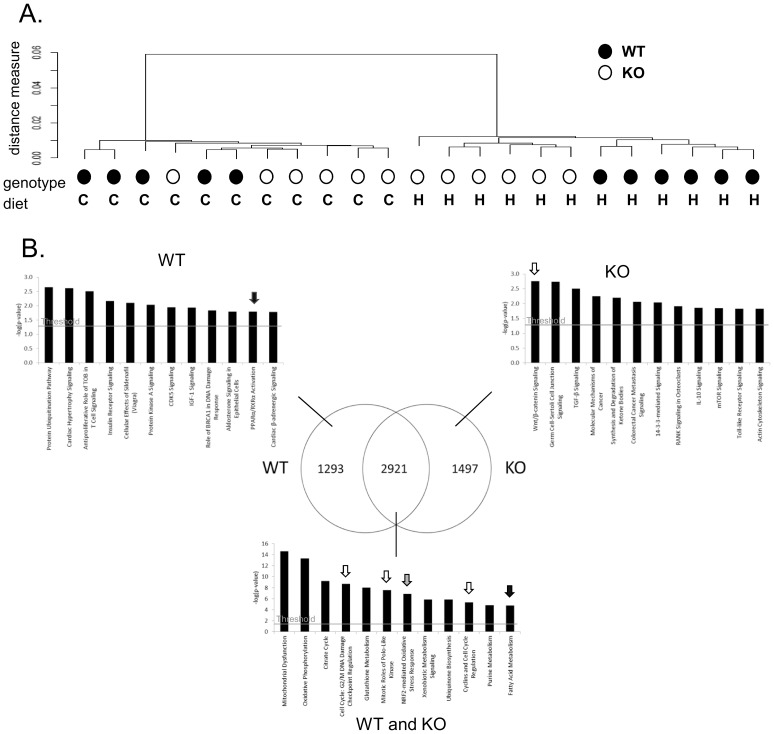
Microarray clustering and pathway analysis of heme-induced differentially expressed genes. **A.** Hierarchical clustering of the microarray data showing that the diet-effect (C = control and H = heme) is more pronounced than the genotype-effect. **B.** Venn diagram showing that 69% of the heme-induced changes (q<0.01 and signal intensity>20 in at least one of the treatments) in WT mice could also be found in KO mice. Ingenuity canonical pathway analysis shows that overlapping genes in Venn-diagram are involved in cell cycle-related processes (open arrows) and Nrf2-mediated oxidative stress response (gray arrow). There is hardly any effect of genotype on fatty acid metabolism-related processes (black arrow). WT mice show PPARα activation (black arrow in WT panel) and KO mice Wnt signaling (white arrow in KO panel). Note that pathways in overlap are much more significant than the WT or KO specific pathways.

**Figure 3:**
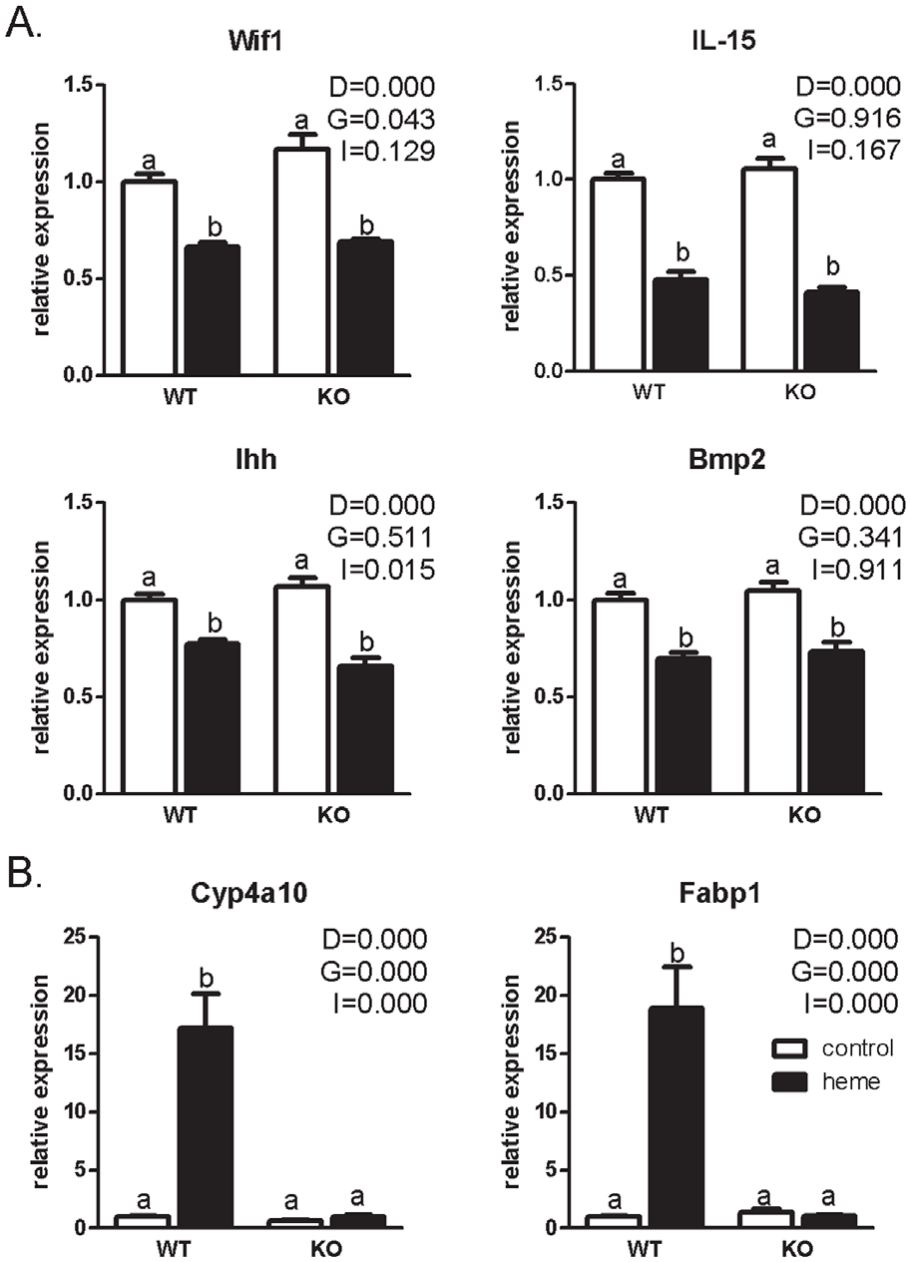
Gene expression of signaling molecules involved in hyperproliferation (A) and of PPARα targets Cyp4a10 and Fabp1 (B). Expression of the WT control group is set to one. Expression of all other groups is relative to WT control. P-values for main effects (D for diet, G for genotype and I for interaction) by a two-way ANOVA are indicated. A and b indicate significant different groups (p<0.05) determined by a Bonferroni post hoc-test.

Although proliferation was not higher in the KO mice compared to the WT on the heme diet, microarrays were performed to determine whether the heme-induced expression of lipid metabolism-related genes and stress-related genes were changed in KO mice compared to WT mice. Hierarchical clustering of the microarray data revealed that the diet-induced effect on gene expression is stronger than the effect of genotype (Figure 2A). Figure 2B shows that there is a pronounced overlap in heme-induced differentially expressed genes between the WT mice and the KO mice. Pathway analysis using the Ingenuity Canonical Pathways program revealed that among the overlapping genes, genes involved in pathways related to cell cycle (open arrows), Nrf2- meditated oxidative stress response (light gray arrow) and lipid metabolism (black arrow) were present. The induction of cell cycle genes in both the WT and the KO is in line with the Ki67 results showing similar hyperproliferation in both heme-fed groups. We verified the expression of our previously identified downregulated feedback inhibitors of proliferation Wif1, IL-15, Ihh and Bmp2 [5]. In the current study, these signaling molecules were similarly downregulated on the heme-diet in both WT and KO mice (Figure 3A). Remarkably, Wnt/β-catenin signaling was the most significant pathway that was changed in the KO mice (Figure 2B, open arrow). Looking at this pathway in more detail revealed that changes in genes in this pathway were not related to changes in cell cycle, but to a 4-fold upregulation of Fosl1 (also called Fra1), which is known to repress the Nrf2-dependent antioxidant response [19], [20].

As expected, pathway analysis showed that changes in PPARα/RXRα activation are restricted to the WT mice (Figure 2B, black arrow). However, a more detailed analysis showed that also in the KO mice still numerous PPARα target genes are regulated by heme, most of them to a similar extent as in WT mice (Figure 1B, the 2^nd^ (WT) and 3^rd^ (PPARα KO) fold-change listed between brackets). Only for Cyp4a10, Fabp1, Acsl1, Slc27a2 and Acaa1a the heme-induced regulation is absent in the PPARα KO mice. For Cyp4a10 and Fabp1, the highest upregulated genes in our previous experiment, expression levels in WT and KO mice are depicted in Figure 3B, showing the lack of a heme-induced upregulation in the KO mice. This indicates that differences in ω-oxidation of fatty acids by Cyp4a10 and in binding hydrophobic lipids by Fabp1 do not affect the heme-induced hyperproliferation. The heme-induced upregulation was thus blocked for five PPARα target genes only, implying that lipid metabolism can still play a role in the heme-induced hyperproliferation, but that this is not dependent on PPARα per se.

### Antioxidant Defense was Compromised in KO Mice on a Heme Diet

**Figure 4:**
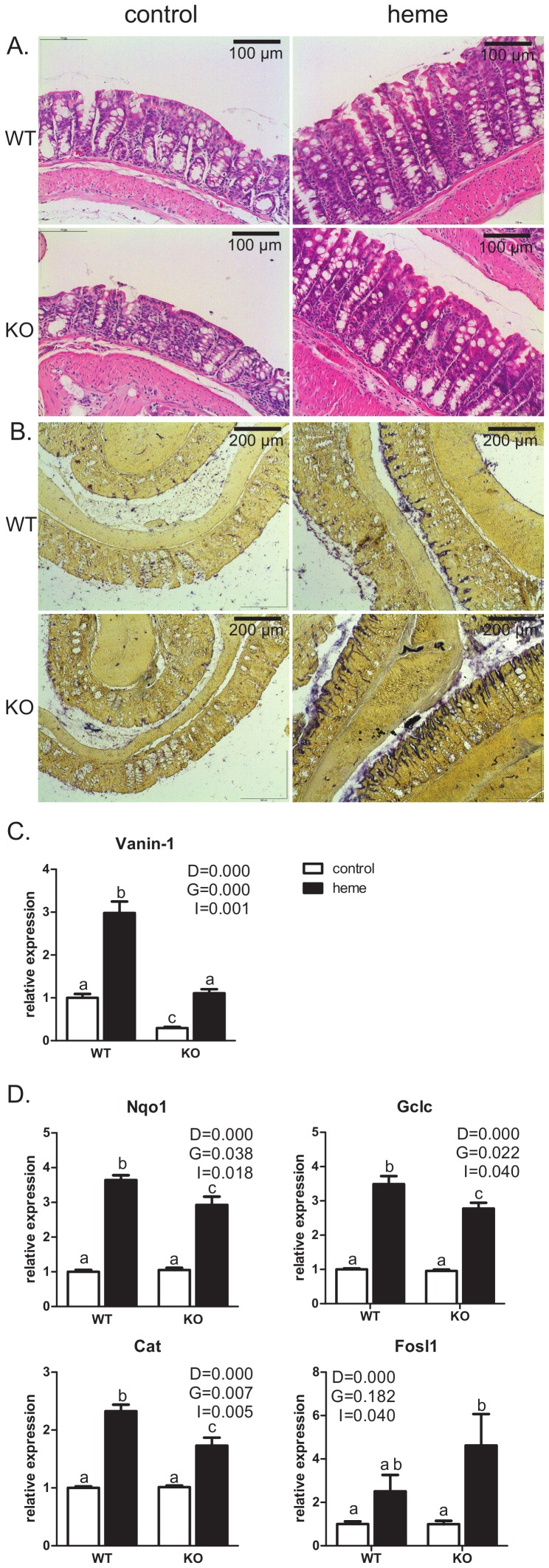
Heme-induced stress response. **A.** Representative pictures of H&E staining of mouse colonic mucosa after 14 days of control- versus heme diet. **B.** Representative pictures of colon tissue stained for Alkaline phosphatase activity, a marker for ROS stress. **C.** Expression of Vanin-1. **D.** Expression of genes involved in antioxidant response Nqo1, Cat, Gclc and Fosl1. Expression of the WT control group is set to one. Expression of all other groups is relative to WT control. P-values for main effects (D for diet, G for genotype and I for interaction) by a two-way ANOVA are indicated. A,b and c indicate significant different groups (p<0.05) determined by a Bonferroni post hoc-test.

As describes above, Figure 2B shows that, both in WT and KO mice, heme changes the NRF2-mediated oxidative stress response. To investigate whether this occurs to a different extent we determined the levels of oxidative stress and damage to the colon tissue in the 4 groups. The overall morphology of the tissue was visualized by an H&E staining (Figure 4A), showing a similar ruffled surface epithelium and deep crypts in the heme-fed WT and KO mice. This indicates similar heme-induced surface injury and luminal necrosis which have been investigated in detail earlier [5], [21]. Alkaline phosphatase activity is a marker of ROS stress [22], and colon sections were stained for alkaline phosphatase activity (Figure 4B). A higher staining intensity was found in the heme-fed groups, which indicated that the ROS stress was higher in these mice compared to the controls. Furthermore, KO mice on a heme diet displayed an even more pronounced staining than WT mice on a heme diet.

Vanin-1 is induced by oxidative stress [23], and was upregulated by dietary heme both in the WT (3-fold) and in the KO mice (4-fold) (Figure 4C), indicating that there is oxidative stress in both WT as well as KO mice on the heme diet. The stress-related induction of Vanin-1 is thus PPARα independent. However, the basal expression level of Vanin-1 was about 3-fold lower in the KO mice compared to the WT, which indicates that the basal vanin-1 levels are controlled by PPARα (also shown in [24]). Other oxidative stress markers, such as expression levels of Metallothionein-1 (Mt1) [25], mast cell hyperplasia [26] (shown by expression levels of e.g. Mcpt1 and 2, Cpa3), Hif1α expression [27] and Mmp9 expression [28] were also explored. These markers showed subtle higher inductions by heme in KO mice compared to WT mice, but these differences did not reach significance (data not shown). Together these data indicated that there is slightly more oxidative stress in the KO compared to the WT mice.

**Figure 5:**
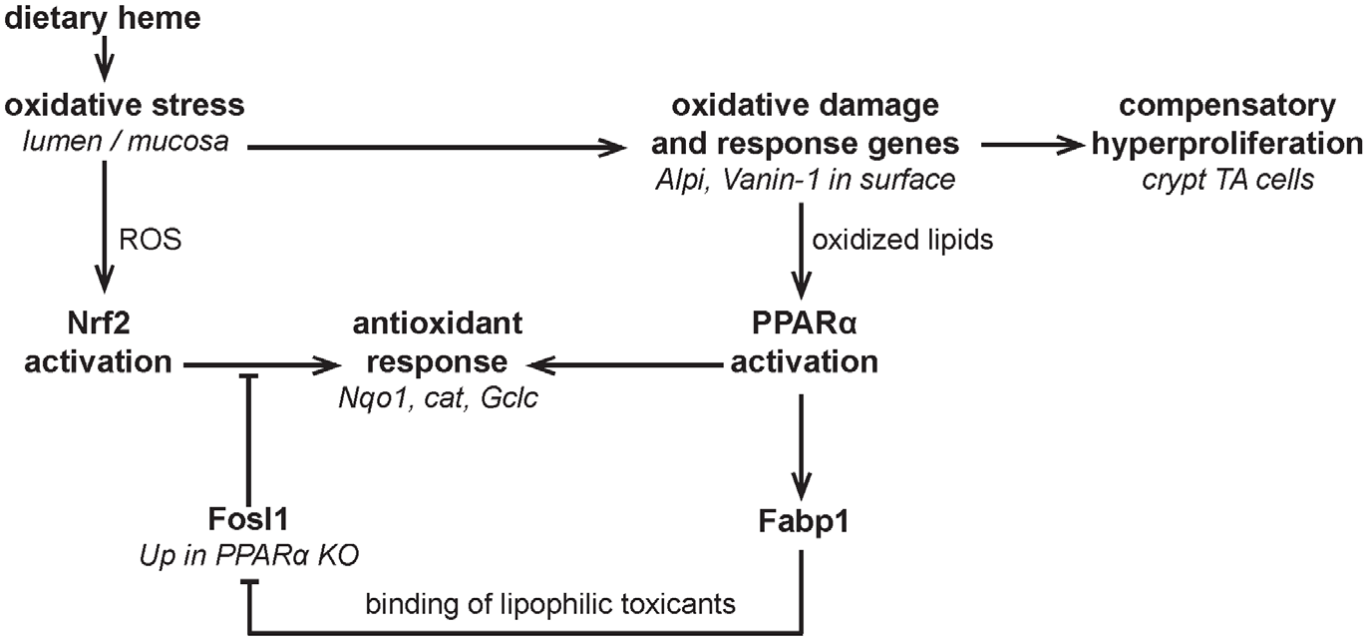
Hypothesized mechanism by which heme induces PPARα and modulates the antioxidant response. Dietary heme induces oxidative stress by generating reactive oxygen species and the production of lipid peroxidation products. ROS induces Nrf2 activation and oxidized lipids activate PPARα leading to an antioxidant response. In PPARα KO mice Fosl1 is upregulated which can inhibit the Nrf2 antioxidant response. Fosl1 upregulation might occur via lipophilic toxicants. The free concentration of these toxicants is probably higher in the KO mice due to the absence of the toxicants-binding Fabp1. As there was no role of PPARα in the heme induced compensatory hyperproliferation of transit amplifying (TA) crypt cells, the question mark indicates a dubious relationship.

Next we determined whether the increased heme-induced oxidative stress in KO animals is due to a compromised antioxidant defense. This implies that a heme-genotype interaction should determine the expression of antioxidant genes. Indeed, antioxidant defense genes show a significant interaction and were induced to a lower extent in the KO mice compared to the WT mice by heme (shown for NAD(P)H quinone oxidoreductase 1 (Nqo1), glutamate-cysteine ligase (Gclc), and catalase (Cat) in Figure 4D. Superoxide Dismutase 1 (Sod1) expression shows a similar pattern although not significant). This shows that the protective response of the mucosa to the heme-induced ROS production was attenuated in the KO mice compared to the WT mice. The significant upregulation of Fosl1 in the KO mice (Figure 4C) could have contributed to the attenuation of these Nrf2-induced antioxidant responses. Together our data indicate that this attenuated defense against oxidative stress in the epithelial surface of the colon does not affect heme-induced hyperproliferation.

## Discussion

This study shows that the transcription factor PPARα does not play a causal role in the heme-induced hyperproliferation and hyperplasia, despite the very high upregulation of PPARα target genes in the colonic mucosa of mice on a heme-rich diet. Lipid metabolism per se can however still play a role in the heme-induced hyperproliferation as most of the lipid metabolism-related genes, including numerous PPARα target genes are still induced in PPARα KO mice on the heme diet. Dietary heme catalyzed the production of ROS which results in the production of oxidized lipids. These oxidized lipids are ligands for PPARα, and this could explain the induction of PPARα target genes in WT mice on the heme diet. It is unlikely that heme itself activates PPARα as planar molecules, such as heme, do not fit in the Y-shaped ligand-binding cavity of PPARα isotypes [29]. Although the differentially expressed genes are well-known PPARα target genes, our results indicate that other transcription factors, e.g. PPARγ or PPARβ/δ, can take over the role of PPARα in the PPARα KO mice [30], [31]. The general expression of PPARγ in colon makes it a reasonable candidate to compensate for the lack of PPARα. PPARγ is previously described to be able to compensate for PPARα in PPARα KO mice [30], however in contrast to these previous findings we did not find a significant upregulation of PPARγ gene expression in the KO mice. This does not rule out a compensatory mechanism by PPARγ per se, as an enhanced activation is not necessarily accompanied by an increased gene expression. Activation of PPARγ was hard to study as the overlap between PPAR target genes is high and currently no specific target genes in colon are known to discriminate between the activation of the different PPARs. A compensatory mechanism by PPARs might explain why lipid-metabolism-related genes are still highly upregulated by dietary heme in the PPARα KO mice.

The five PPARα target genes of which the induction by heme was blocked in the PPARα KO animals were Fabp1, Cyp4a10, Ascl1, Slc27a2 and Acaa1a. Therefore, these genes do not play a role in the heme-induced hyperproliferation. The exposure of the epithelial surface to lipid peroxidation products and to cytotoxic molecules in the WT and KO heme-fed mice must be similar as TBARS and cytotoxicity measurements show no differences between WT and KO heme-fed mice. However, there is more oxidative damage in surface cells of the KO mice, as shown by the alkaline phosphatase activity staining and Vanin-1 induction. In line with this, there is a reduced antioxidant defense in the KO mice, which is reflected by the lower induction of Nrf2 target genes, such as Nqo1 and Cat, upon heme feeding in the KO mice. Together these data suggest that there is less protection against oxidative stress and/or lipid peroxidation in the heme-fed KO mice, indicating that PPARα plays a protective role in the heme-induced oxidative stress response. This attenuated antioxidant response did not lead to an increased cell proliferation as hypothesized, and it is therefore unlikely that oxidative stress induces signaling to the crypt to initiate hyperproliferation. It is more plausible that cytotoxic stress induces hyperproliferation and hyperplasia in the colon of heme-fed mice. We have shown earlier that dietary antioxidants prevent all detrimental effects of dietary heme in the colon [32]. However, that study could not differentiate between causal effects of oxidative and cytotoxic stress, because heme induced cytotoxicity was also prevented by antioxidants. This is consistent with other studies [4], [21] showing that cytotoxicity is due to a covalently modified porphyrin formed from heme, probably by radical-mediated addition reactions in the gastrointestinal tract. It can be speculated that this complex radical-mediated formation of the cytotoxic heme factor lags behind the instantaneous generation of oxygen radicals by heme. Whether this is the case requires investigations of the possible differential time course of the heme-induced oxidative and cytotoxic stress. Such a study is now in progress.

The induction of Vanin-1 is slightly higher in the KO heme-fed mice compared to the control heme-fed mice (4-fold vs. 3-fold). This might suggest that there is slightly more oxidative stress in the KO mice, which could be the result of a lower antioxidant response in the KO mice. From our previous study [5] in which we separated colonic surface and crypt cell gene expression, expression of Vanin-1 was 2 times higher at the surface epithelium compared to the crypt under control conditions. Upon heme feeding, Vanin-1 expression increased 3-fold at the surface epithelium, while the expression remained unchanged in the crypt (results can be found in the Gene Expression Omnibus, accession number GSE27849). This implies that the oxidative stress is exclusively induced at the surface epithelium. Besides its role as oxidative stress marker, Vanin-1 is recently proposed as a causal factor in colonic hyperproliferation [33]. As mentioned above, the expression of Vanin-1 is induced in the surface epithelium and proliferation occurs from the stem cells in the crypt. This implies that if Vanin-1 plays a role in the heme-induced hyperproliferation Vanin-1 should signal from the surface to the crypt to initiate this hyperproliferation. This is not supported in this study, as the levels of Vanin-1 expression are 3 times higher in WT heme-fed mice compared to KO heme-fed mice while proliferation rates are similar. Thus, in our study we could not correlate the gene expression level of Vanin-1 to the level of proliferation in the colon.

Nrf2 is the prominent transcription factor that regulates the antioxidant response. Nrf2 is essential for the antioxidant response element (ARE)-mediated induction of many cytoprotective enzymes, such as Cat and Nqo1. Nrf2 activity is controlled by Keap1. Oxidative stress (generated e.g. by heme-rich diet) can oxidize critical cysteine residues in Keap1, resulting in inactivation of Keap1 and accumulation of Nrf2 in the nucleus, where it binds to ARE in the promoter region of many antioxidative genes, initiating their transcription (reviewed in [34]). Although the antioxidant response is predominantly regulated by Nrf2, there is an overlap in target genes between this transcription factor and PPARα [9], [18]. Bunger et al. [18] showed that in the intestine, known Nrf2-target genes Cat and glutathione-related genes, are also regulated by PPARα. This might explain the lower induction of Cat and glutathione-related genes in the heme-fed KO mice compared to the heme-fed WT mice. However, as other antioxidant response genes, such as Sod1 and Nqo1 are no PPARα target genes an additional mechanism must be present by which PPARα indirectly influences the Nrf2-driven antioxidant response. This possible additional mechanism by which PPARα can be protective involves regulation of Fosl1. Fosl1 is significantly upregulated only in the PPARα KO mice. Fosl1 represses the Nrf2-dependent expression of antioxidant response element (ARE) containing genes such as Nqo1 and Gclc [19], [20]. How PPARα influences Fosl1 expression is largely unknown. Direct regulation of Fosl1 by PPARα is not likely because its expression is not modulated by the PPARα-specific ligand WY14643 [18]. We propose that Fatty Acid binding protein 1 (Fabp1) acts as an intermediate in the PPARα-dependent regulation of Fosl1. In contrast to Fabp2, Fabp1 has a large hydrophobic pocket and can bind toxic hydrophobic molecules such as (oxidized) long chain fatty acids, bile acids and heme [35]. There is a heme-induced upregulation of Fabp1 expression in the WT mice, but this induction is blocked in the KO mice. The absence of Fabp1 in the KO might lead to more unbound hydrophobic toxicants in colon cells of these KO mice. Fosl1 can be induced by toxic compounds [36], and we hypothesize that unbound toxicants present in the KO mice can induce the expression of Fosl1. The expression of Fabp2 was induced by heme in both the WT and the KO with 1.5-fold, but Fabp2 cannot bind toxic molecules such as heme in its small pocket. It is therefore unlikely that Fabp2 plays a role in the induction of Fosl1. The hypothesized mechanism suggests that PPARα plays its protective role in the colon via its target Fabp1 which can bind large hydrophobic molecules and thereby preventing the induction of Fosl1 (Figure 5). This mechanism predicts that Fabp1 KO mice should have higher Fosl1 levels and an attenuated antioxidant response compared to WT mice. Whether this is the case requires further investigation, but our mechanism is corroborated by studies showing that cells transfected with Fabp1 have lower intracellular ROS levels and a reduction of oxidative stress compared to untransfected cells [37], [38].

The PPARα KO and WT mice have a SV129 background. Our previous studies carried out with heme-rich diets were performed in C57Bl6J mice and we see similar effects on luminal cytotoxicity and epithelial proliferation. Besides proliferation, also similar effects were found on gene expression as similar genes were induced in the SV129 WT heme compared to the C57Bl6J (Pearson correlation coefficient of 0.881, n = 3673 genes). This shows that there is a similar response to dietary heme in these two mouse stains. These results are also similar to results observed in rats [21], indicating that the heme effect is species and strain-independent.

Taken together, we conclude that the heme-induced hyperproliferation is not mediated by PPARα. As only 5 PPARα target genes did not respond to heme in the KO mice, a possible role in the heme induced hyperproliferation for other PPARα target genes and lipid metabolism-related genes in general cannot be excluded. Our data do suggest that PPARα plays a protective role against oxidative stress induced by dietary heme in the colonic epithelial cells. Moreover, our results indicate that most probably not ROS-induced stress, but cytotoxicity-induced stress initiates colonic hyperproliferation.
